# Priming innate immunity and long-term outcome

**DOI:** 10.1186/s40348-026-00244-1

**Published:** 2026-05-27

**Authors:** Victoria Pradler, Clyde J Wright, Nazanin Kazemi-Butterfield, Kirsten Glaser

**Affiliations:** 1 Department of Pediatrics, Icahn School of Medicine at Mount Sinai, New York, NY USA; 2 Medical Faculty, University of Muenster, Muenster, Germany; 3 Department of Pediatrics, Section of Neonatology, Children’s Hospital Colorado, University of Colorado School of Medicine Aurora, Aurora, CO USA; 4 Division of Neonatology, Department of Women’s and Children’s Health, University of Leipzig Medical Center, Liebigstrasse 20a, Leipzig, 04103 Germany

**Keywords:** Preterm infants, Inflammation, Multiple-hit sequence, Innate immunity, Immune priming, Inflammatory signaling, Neonatal morbidity, Long-term outcome

## Abstract

**Background:**

While advancements in neonatal intensive care have significantly improved the survival of preterm infants, inflammation-related complications continue to be a major factor in short- and long-term morbidity, especially in the most immature babies. Profound evidence indicates that prenatal and postnatal inflammatory exposures, interacting in a multi-hit sequence, significantly affect both immune responses and long-term organ development.

**Content:**

While preterm birth frequently represents the initial trigger of an adverse cascade of inflammation, various postnatal environmental factors, such as respiratory support, oxygen therapy, and neonatal infections, contribute to this process, with each event serving as an inflammatory stressor independently associating with inflammation-driven tissue damage. The preterm innate immune system seems particularly susceptible not only to infection, but to pro-inflammatory immune responses and sustained immune activation. Mechanistically, several processes have been implicated, including disturbed homeostasis of inflammatory mediators, antioxidant enzymes, and proteases, as well as altered pathogen recognition, and particularities in the resolution of inflammation. In addition, trained immunity, immune tolerance, epigenetic and metabolic reprogramming, and interactions between altered gut microbiota and the developing immune system may further shape these responses. Subsequently altered, often increased or sustained inflammation has been recognized as a central mechanism linking early-life exposures to impaired lung, brain, gut, and retinal development, and adverse long-term outcomes. The frequency of episodes of inflammation seems to significantly impact the latter.

**Conclusions:**

A better understanding and greater awareness may enable improved risk stratification and avoidance of inflammatory exposures, and promote the development of more targeted strategies to prevent adverse inflammation during a vulnerable period.

## Inflammatory exposures in preterm infants: from drivers of preterm birth to postnatal hits

Therapeutic advancements in neonatal intensive care have led to substantial improvements in the survival of preterm infants. Yet, the incidence of major complications associated with prematurity, including bronchopulmonary dysplasia (BPD), preterm white matter disease (WMD), necrotizing enterocolitis (NEC), and retinopathy of prematurity (ROP), remains high – particularly among infants born at extremely low gestational ages. Substantial evidence indicates that inflammation plays a central role in the pathogenesis of all these morbidities.

### Prenatal inflammatory exposures

Preterm infants are frequently exposed to inflammatory stimuli, both before and after birth. In fact, preterm birth itself may represent the first event in a cascade of inflammatory and developmental perturbations that shape immune function and organ maturation. One of the major drivers of preterm birth is intrauterine inflammation, most commonly manifesting as chorioamnionitis. Histologic chorioamnionitis complicates as much as up to 70% of spontaneous preterm births, with the lowest gestational ages being mostly affected. In contrast, it is present in fewer than 30% of term deliveries [1]. In addition, there is a growing recognition that pregnancies complicated by conditions such as intrauterine growth restriction and preeclampsia are characterized by sterile inflammatory signaling within the placenta [2]. These early exposures may culminate in a fetal systemic inflammatory response of variable degree and shape subsequent immune responses to secondary insults.

### Postnatal inflammatory hits in the neonatal intensive care unit

Postnatally, interventions inherent to neonatal intensive care as well as infectious exposures may initiate or amplify inflammatory signaling in preterm infants [3]. Common inflammatory stressors include mechanical ventilation, supplemental oxygen exposure, neonatal infection, nutritional support, and transfusion of blood products. Within a multiple-hit framework, these exposures may interact additively or synergistically with ongoing organ and immune system development during a critical window of vulnerability [3–5]. Mechanical ventilation is a major contributor to lung injury through mechanisms including volutrauma, barotrauma, atelectrauma, and biotrauma, all of which promote pulmonary and systemic inflammatory responses. However, supplemental oxygen itself also represents an important hit to the immature lung, inducing oxidative stress, epithelial and endothelial injury, inflammatory cell recruitment, and activation of pro-inflammatory cytokine pathways that may impair alveolar and vascular development independently of mechanical ventilation [5]. Experimental and clinical studies suggest that both ventilator-induced injury and oxygen toxicity contribute to sustained inflammatory activity associated with BPD development [3, 4, 6] – reflected, for instance, by increased levels of pro-inflammatory cytokines and chemokines [7]. Detection of these biomarkers in preterm tracheal aspirates, blood, and urine seems to correlate with an increased risk of developing BPD [3, 6]. Pro-inflammatory biomarkers have also been associated with the development and disease progression of WMD, NEC, and ROP [4].

### Sustained inflammation and innate immune priming

Whilst neonatal infections are the strongest driver of an inflammatory response, various research groups, including several publications from the multicenter ELGAN study (*Extremely Low Gestational Age Newborn Study*), have measured elevated serum levels of pro-inflammatory biomarkers in the blood or tracheal secretions of preterm infants outside of episodes of infection – very early in the postnatal period and at later, pre-selected time points [8]. Based on these data, the authors discuss the presence of ongoing or sustained inflammation in preterm infants following prenatal and postnatal exposures [8]. Although these findings suggest the presence of inflammation – that is not necessarily apparent – and point to a heightened vulnerability for a secondary and tertiary hit in some infants, it remains unclear whether the elevated inflammatory markers seen at two or more intervals indicate repeated inflammatory episodes or ongoing, unresolved inflammation.

Taken together, these exposures may not only act as sequential inflammatory hits but also functionally reprogram the developing immune system. This process, referred to as innate immune priming, shapes the magnitude and quality of subsequent immune responses and may thereby influence later disease susceptibility and outcomes. In contrast to previous reviews focusing primarily on individual inflammatory diseases or isolated inflammatory pathways, this mini-review integrates the concepts of repetitive inflammatory exposures, sustained innate immune activation, and microbiota-driven immune conditioning as interconnected mechanisms contributing to adverse organ development and long-term outcome in preterm infants.

## Particularities of innate immune responses in preterm infants and mechanisms of innate immune priming

Host-related factors – most notably the degree of developmental immaturity – play a central role in shaping the preterm immune response. Preterm infants are at increased risk of infection, largely due to quantitative and qualitative characteristics of the developing immune system. Moreover, observational and experimental studies indicate an increased susceptibility of preterm neonates to imbalanced and sustained inflammation – attributable, most likely, to unique functional characteristics of the preterm immune system, including differences in immune cell capacities, pattern recognition, and inflammatory signaling, as well as capacities for immune priming [4, 9].

While adaptive cell responses are still not fully mature and might be less functional, preterm infants mainly rely on the non-specific innate immunity for immune defense. Neutrophils and mononuclear cells are key innate immune cells and regulators of inflammation, serving as the first responders. The derived cytokines, chemokines, and reactive oxygen species determine immediate host defense and prime adaptive immune responses. While observational data point out that preterm and term infants exhibit strong pro-inflammatory responses to infection [4, 10], data from experimental studies in neonatal neutrophils and mononuclear cells are partly inconsistent in terms of phagocytic activity and inflammatory capacities. Several studies point to gestational-age-dependent patterns [11]. Yet, many experimental studies, including double-hit models using hyperoxia or hypoxia and pathogen exposure, have demonstrated pronounced pro-inflammatory responses compared to adult controls [12]. Additional transcriptomic analyses indicate a significant upregulation of pro-inflammatory signaling pathways in preterm monocytes [7].

This signaling is initiated through the recognition of pathogen-associated and damage-associated molecular patterns (PAMPs and DAMPs) by pattern-recognition receptors (PRRs), such as toll-like receptors (TLRs), NOD-like receptors, RIG-I-like receptors, cyclic guanosine monophosphate adenosine monophosphate synthase-like receptors, and C-type lectin receptors [9]. Particularities of PRR signaling, including the downstream activation of transcription factors (nuclear factor kappa-light-chain-enhancer of activated B cells, NFκB; activator protein 1, AP-1; and the interferon response factors, IRFs), have been linked to pronounced inflammatory responses in neonates. Yet, existing data on pathogen recognition in preterm infants from in vitro and ex vivo studies are inconsistent. Some studies have reported impaired pro-inflammatory responses and reduced phosphorylation of TLR signaling molecules, like p38, ERK1/2, and NF-κB, while others have found similar or heightened inflammatory responses and equal or increased phosphorylation levels, depending on cell type and context [9, 11–13]. Altered PRR expression and/or PRR signaling in preterm infants has been discussed to promote exaggerated inflammation [11]. In addition, gestational-age-dependent particularities in the activation and degradation of NF-κB subunits may result in prolonged inflammatory signaling [14].

Despite mounting significant pro-inflammatory responses, preterm infants might not be able to balance this with adequate anti-inflammation. In a healthy organism, the innate immune response serves to support balanced cytokine responses and limit and resolve inflammation. Mechanisms involved in immune homeostasis and the termination of inflammation seem to be altered in neonates: Counterbalancing responses appear comparatively diminished and functionally immature, and impairments in phagocyte activity, delayed apoptosis of inflammatory cells, and prolonged survival of neonatal neutrophils have been reported [10, 13]. Moreover, adenosine, cAMP, and S100 alarmins, as well as epigenetic and post-transcriptional regulations, have been discussed as contributing mechanisms [10, 11]. Most recently, altered expression of immune checkpoint molecules on neonatal versus adult T cells has been associated with an impaired ability to terminate inflammation [15]. In summary, these features may bear the risk of deleterious effects of imbalanced and exaggerated or sustained inflammation. Consequently, an inflammatory state may persist, worsen, or be newly initiated during neonatal intensive care, accounting for the development of inflammation-related disorders, such as BPD, WMD, NEC, and ROP [4]. Moreover, inadequate anti-inflammatory responses may lead to an increased risk of infection-related morbidity and mortality in preterm neonates.

A large body of evidence suggests that immunological memory is not an exclusive hallmark of the adaptive immune response and that the exposure to an immune stimulus may induce persistent functional reprogramming of innate immune cells, impacting responses upon subsequent exposures to the same or a different stimulus [16, 17]. The concept of trained immunity describes long-term functional reprogramming of innate immune cells, and in some settings their progenitors, after exposure to exogenous PAMPs or endogenous DAMPs, resulting in a prolonged or enhanced response to a subsequent hit after return to a non-activated state. Clinically, preexposure of innate immune cells, such as monocytes, to a wide range of infectious agents may trigger an exaggerated inflammatory response upon a secondary infection. This program seems mediated by coordinated epigenetic and metabolic remodeling. Functionally, trained innate immune cells commonly express higher levels of pro-inflammatory mediators, such as Interleukin (IL-) 1β, tumor necrosis factor (TNF-) α, IL-6, as well as reactive oxygen species [16, 17]. In terms of evolutionary biology, a more robust innate immune response on reinfection with the same pathogen or upon infection with an unrelated one may provide enhanced protection [16]. In preterm infants, these mechanisms may contribute to increased inflammatory responses. Conversely, perinatal infection and inflammation, as in chorioamnionitis, may also result in a temporary state of reduced immune cell responsiveness to a secondary pathogen – by functional reprogramming [11]. This condition, referred to as endotoxin tolerance, is characterized by selective hyporesponsiveness with reduced induction of pro-inflammatory genes rather than global immune paralysis [17]. Consistent with this tolerance-like state, exposure to chorioamnionitis has been associated with altered preterm monocyte responses, including reduced HLA-DR expression and downregulation of genes involved in antigen presentation and inflammasome signaling [18]. The resulting tolerance-like state may increase the susceptibility to infection and subsequent inflammation in preterm infants.

Both training and tolerance reactions seem to be shaped by alterations in PRR-associated signaling and triggered by the individual PAMP/DAMP or the dose of exposure, respectively. In addition, exposure to individual pathogens has been discussed as a key factor shaping neonatal immune response [10]. While the stressor-dependent hypothesis suggests that the respective PAMP/DAMP, such as b-glucan and BCG, trigger the induction of trained immunity versus tolerance, the dose-dependent hypothesis speculates that high-dose versus low-dose exposure makes the difference. Thereby, several PAMPs, such as b-glucan and BCG, as well as low-dose priming, have been associated with the induction of trained responses, whereas exposure to Gram-negative endotoxin and high-dose priming have been related to the development of immune tolerance to a secondary hit [16, 17].

## Early-life microbiota and immune priming

Over the past decade, the role of the early-life microbiota as a central regulator of immune development and organ function has become increasingly evident, and concepts like the gut-lung, gut-brain, and gut-retina axes have gained prominence. These refer to bidirectional interactions mediated by microbial metabolites, immune signaling pathways, altered T-cell responses, and neuroimmune mechanisms [19, 20]. Germ-free animal models confirm this central role of the microbiome in early immune maturation [21]. Recent systems-immunology studies demonstrated that preterm immune development follows dynamic postnatal trajectories driven by microbial interactions and nutritional exposures, while early gut dysbiosis may interfere with normal immune maturation [22]. Preterm infants are particularly susceptible to gut dysbiosis and exhibit characteristic alterations in the microbial composition in comparison to term infants, including reduced microbial diversity, a predominance of facultative anaerobes like *Enterobacteriaceae* and *Enterococci*, and a delayed colonization by obligate anaerobes such as *Bifidobacterium* and *Bacteroides* [20, 23]. These patterns may reflect physiologic immaturity and are further shaped by antenatal and postnatal factors, including maternal and fetal infections, antibiotic exposure, mode of delivery, delayed enteral feeding, parenteral nutrition, impaired gut motility, and the hospital microbial environment [20, 23]. Moreover, altered PRR signaling at mucosal surfaces might limit microbial diversity, potentially favoring the colonization of pathogenic microorganisms [24]. Human studies further support a central role of microbial and nutritional exposures in shaping immune maturation in preterm infants. Infants receiving their mother’s own milk showed a more similar immune development to term-born infants than those receiving donor milk – an effect that may involve additional bioactive milk components [25]. Likewise, supplementation with *Bifidobacterium* in preterm infants has been associated with reduced pathobiont abundance and increased fecal acetate and lactate concentrations, suggesting that microbial metabolomic remodeling may influence the intestinal inflammatory milieu [26]. Extending these metabolomic observations, a systems-level human study demonstrated that depletion of *Bifidobacterium* and impaired utilization of human milk oligosaccharides are associated with inflammatory immune dysregulation in early life [27]. Similarly, intestinal *Klebsiella* overgrowth has been associated with pro-inflammatory immune signatures, altered γδ T-cell responses, and subsequent brain injury in preterm infants, further supporting the concept of a gut–microbiota–immune–brain axis in prematurity [19]. Mechanistically, early-life gut dysbiosis may impair intestinal barrier integrity, alter the production of key microbial metabolites such as short-chain fatty acids and bile acids, and modulate immune responses through altered PRR signaling and downstream inflammatory pathways [20]. Clinically, disruptions in the development of the intestinal microbiome and microbiota-immune interactions have been associated with increased susceptibility to inflammatory morbidities in preterm infants, including NEC, BPD, and late-onset sepsis [20, 28]. These relationships are bidirectional, as inflammatory conditions and intensive care interventions, such as oxygen therapy and infection, can further perturb the developing microbiome, creating a self-perpetuating cycle of dysbiosis and inflammation. Notably, an increasing number of studies have shed light on the role of individual gut commensals and gut microbiota-derived extracellular vesicles, in particular, in shaping the immune response to an infectious stimulus by inducing trained effects in innate immune cells [29].

## Impact on short- and long-term outcomes

Prenatal and postnatal inflammatory exposures in preterm infants have been associated with adverse outcomes across different developmental stages, ranging from neonatal morbidities to impaired neurodevelopment and organ dysfunction later in childhood. Several meta-analyses have demonstrated that exposure to histologic chorioamnionitis is associated with a significantly increased risk of BPD in preterm infants [30]. Moreover, intrauterine inflammation and/or the presence of microbial pathogens in the placenta have been linked to adverse neurological development, including an increased risk of cerebral palsy and impaired neurocognitive function persisting into school age [31, 32]. Notably, detection of potentially anti-inflammatory microorganisms such as *Lactobacillus species* in the placenta may be associated with a lower risk of impaired long-term neurocognitive functions in preterm infants [32]. Postnatal inflammatory exposures can, in turn, initiate or further amplify early immune perturbations. Among these, neonatal sepsis represents one of the most potent triggers of systemic inflammation. Multiple cohort and network studies have demonstrated that preterm infants with a history of neonatal sepsis carry an increased risk of developing BPD [5]. In addition to pulmonary complications, neonatal sepsis has been associated with neurological injury, including periventricular leukomalacia and impaired neurodevelopment [33, 34]. Numerous large cohort studies, such as the German Neonatal Network, have shown that recurrent episodes of sepsis are particularly associated with poor outcomes [34, 35]. Preterm infants with recurrent episodes of LOS had a significantly increased risk of treatment-warranted ROP [35], impaired psychomotor development, and significantly reduced cognitive performance during childhood. Infants with a history of repeated sepsis episodes showed a three-fold increased risk of adverse motor development [34]. Experimental studies support a causal role of fetal and neonatal systemic inflammation in adverse long-term neurologic outcomes. In animal models of neonatal sepsis, induction of inflammation via intraperitoneal injection of lipopolysaccharide resulted in impaired learning performance weeks after the insult [36]. The observed deficits were paralleled by increased systemic inflammatory mediators as well as elevated expression of pro-inflammatory cytokines in the hippocampus, suggesting that systemic inflammation may directly affect brain development [36].

In addition to fulminant septic conditions, clinically inapparent persistent inflammation in the first weeks of life seems strongly associated with adverse long-term outcomes. Within the ELGAN study cohort, preterm infants with elevated levels of pro-inflammatory cytokines and chemokines persisting for two weeks or longer were at increased risk of severe BPD as well as microcephaly, WMD, and impaired neurodevelopmental scores at two years of age [8]. Mechanistic studies further support the concept that early immune dysregulation contributes to adverse long-term outcomes in preterm infants. Alterations in monocyte subset composition have been associated with the later development of BPD in infants born before 32 weeks of gestation [7]. In particular, an early expansion of intermediate monocytes accompanied by increased intracellular expression of TNF-α has been observed in infants who subsequently developed BPD [7]. Complementary transcriptomic analyses revealed an upregulation of inflammatory signaling pathways as well as pathways associated with reactive oxygen species production and oxidative stress in these infants [7]. Supporting a link between early-life microbial dysbiosis and inflammation-driven organ injury, a systematic review covering six studies on preterm airway microbiome and later development of BPD in infants born before 32 weeks of gestation found reduced microbial diversity, as well as early and persistent dysbiosis to be associated with an increased risk of chronic lung disease [28]. Some studies also suggested an association with Ureaplasma colonization [28]. In addition, emerging evidence sugests that early-life dysbiosis may contribute to persistent respiratory morbidity extending beyond infancy, including recurrent infections, wheezing disorders, and asthma later in childhood [37].

## Therapeutic and preventive implications

Considering the pivotal involvement of inflammation in the pathogenesis of neonatal morbidities and adverse long-term outcomes in preterm infants, a central challenge for neonatal medicine is therefore to identify infants at the highest risk for persistent inflammation and to develop strategies that mitigate secondary and tertiary inflammatory insults. Potential approaches include improved prevention of infectious complications, beneficial modulation of the developing microbiome, and refined risk stratification strategies enabling targeted therapeutic interventions. However, substantial knowledge gaps remain. The complex interplay between prenatal exposures, postnatal environmental factors, genetic susceptibility, and developmental windows of vulnerability likely differs between individual infants, making prediction of long-term outcomes challenging. The use of systemic corticosteroids to prevent BPD exemplifies the complexity of the therapeutic goal of attenuating inflammation to improve neonatal outcomes. Through wide-ranging genomic and non-genomic mechanisms, steroids dampen inflammation. Early reports confirmed the effectiveness of glucocorticoids, specifically dexamethasone, in preventing BPD in those extremely high-risk neonates requiring prolonged mechanical ventilation and supplemental oxygen. Yet, in further, early glucocorticoid exposure (within the first week) was found to increase the risk of cerebral palsy and poor neuromotor and cognitive outcomes [38]. Considering the clinical benefits and risks, a 2024 Cochrane overview identified late dexamethasone, beyond day 7, as the only effective strategy for postnatal systemic corticosteroids, still requiring careful selection [38]. Nevertheless, the promise of interrupting the inflammatory process to improve outcomes, while minimizing unwanted adverse effects, leaves neonatologists searching for improved and more targeted approaches [3]. In this context, macrolides, particularly azithromycin, have been discussed as potential alternative agents due to their anti-infective and anti-inflammatory properties and due to beneficial effects seen in children and adults with inflammatory lung diseases. However, the recently published AZTEC trial (*Azithromycin Therapy for Chronic Lung Disease of Prematurity*) did not find reduced rates of BPD in infants born < 30 weeks’ gestation prophylactically treated with azithromycin in the first days of life, independent of their *Ureaplasma* colonization status, thus discouraging routine use [39]. Finally, modulating pro-inflammatory signaling downstream of transcriptional expression may be a promising strategy for attenuating inflammatory responses and subsequent tissue injury. The IL-1 receptor antagonist Anakinra is currently undergoing evaluation in a phase I/II clinical trial to determine its safety in infants born between 24 + 0 and 27 + 6 weeks of gestation. Data are not yet available [40]. A major challenge for a more targeted prevention and tailored treatment is the delay between the early vulnerable period in the first weeks of life – when infants face high pro-inflammatory exposure and strong risk for dysbalanced and sustained inflammation – and the gradual onset of long-term impairments later in life. In this regard, advances in our understanding of how the gut microbiome affects preterm immune responses and organ development may hold a key to new solutions. Deeper insight into microbiota-driven immune programming may enable novel strategies for risk stratification and targeted interventions early in life, including microbiome-modulating approaches such as optimized nutrition, antibiotic stewardship, and the use of pre-, pro-, or synbiotics in the prenatal, perinatal, and postnatal period [41, 42]. Notably, profound data show that the immune properties in human milk change during lactation and impact the protective effect of breastfeeding on both microbiome development and immune maturation [41]. Recent experimental findings indicate that selected microbiota-derived metabolites and defined metabolite-producing commensals, respectively, may represent promising future postbiotic treatment strategies [29].

## Conclusions and future directions

There is profound evidence that prenatal and postnatal inflammatory hits initiate and perpetuate a state of recurrent or sustained immune activation in preterm infants that profoundly affects organ development and neonatal long-term outcome. Thus, effectively targeting inflammation remains a primary therapeutic objective to alleviate the burden of morbidities associated with preterm birth. Ongoing efforts include pharmacological interventions aimed at mitigating adverse intrauterine and postnatal infection and inflammation and at preventing adverse long-term outcomes. However, current management strategies remain limited. Advancing our understanding of the functional characteristics of the preterm immune system, the interrelationships between inflammatory exposures and host immune responses, and their contributions to disease development, is essential for identifying targetable pathogenic mechanisms, minimizing unintended effects, and preserving the beneficial aspects of the innate immune system. Finally, the impact of microbiota composition and the potential role of microbiota-driven immune programming continue to be investigated through clinical and experimental studies.

The integration of repetitive inflammatory exposures, innate immune priming, and microbiota-driven immune conditioning may provide a more comprehensive framework for understanding the heterogeneity of inflammation-associated outcomes in preterm infants. Ultimately, a better understanding and greater awareness enabling improved identification and adequate stratification of at-risk preterm infants is a necessary step forward.

## Data Availability

No datasets were generated or analysed during the current study.

## Abbreviations

BPD: Bronchopulmonary dysplasia
DAMP: Damage-associated molecular pattern
IL: Interleukin
NEC: Necrotizing enterocolitis
NOD: Nucleotide-binding oligomerization domain-containing protein
PAMP: Pathogen-associated molecular pattern
PRR: Pattern recognition receptor
RIG: Retinoic acid-inducible gene
ROP: Retinopathy of prematurity
TLR: Toll-like receptor
TNF-α: Tumor necrosis factor α
WMD (Preterm): white matter disease (WMD)
